# The p14ARF Alternate Reading Frame Protein Enhances DNA Binding of Topoisomerase I by Interacting with the Serine 506-Phosphorylated Core Domain

**DOI:** 10.1371/journal.pone.0058835

**Published:** 2013-03-26

**Authors:** Keya Bandyopadhyay, Pingchuan Li, Ruth A. Gjerset

**Affiliations:** Torrey Pines Institute for Molecular Studies San Diego, California, United States of America; Florida International University, United States of America

## Abstract

In addition to its well-characterized function as a tumor suppressor, p14ARF (ARF) is a positive regulator of topoisomerase I (topo I), a central enzyme in DNA metabolism and a target for cancer therapy. We previously showed that topo I hyperphosphorylation, a cancer-associated event mediated by elevated levels of the protein kinase CK2, increases topo I activity and the cellular sensitivity to topo I-targeted drugs. Topo I hyperphosphorylation also increases its interaction with ARF. Because the ARF−topo I interaction could be highly relevant to DNA metabolism and cancer treatment, we identified the regions of topo I involved in ARF binding and characterized the effects of ARF binding on topo I function. Using a series of topo I deletion constructs, we found that ARF interacted with the topo I core domain, which encompasses most of the catalytic and DNA-interacting residues. ARF binding increased the DNA relaxation activity of hyperphosphorylated topo I by enhancing its association with DNA, but did not affect the topo I catalytic rate. In cells, ARF promoted the chromatin association of hyperphosphorylated, but not basal phosphorylated, topo I, and increased topo I-mediated DNA nicking under conditions of oxidative stress. The aberrant nicking was found to correlate with increased formation of DNA double-strand breaks, which are precursors of many genome destabilizing events. The results suggest that the convergent actions of oxidative stress and elevated CK2 and ARF levels, which are common features of cancer cells, lead to a dysregulation of topo I that may contribute both to the cellular response to topo I-targeted drugs and to genome instability.

## Introduction

The p14 Alternate Reading Frame (ARF) protein is a cancer-associated protein that plays a well-characterized role in activating the p53 tumor suppressor pathway. ARF is present in normal cells at low to undetectable levels but accumulates in response to oncogene activation [1]–[6]. High ARF levels provide a barrier to oncogene-driven cellular hyperproliferaton by stabilizing wild-type p53 protein and promoting p53-mediated cell cycle arrest, senescence, or apoptosis [6]–[8]. p53 is frequently inactivated in cancer, which disables the p53-dependent tumor suppressor activity of ARF but leaves ARF available to interact with a variety of other proteins, including topoisomerase I (topo I) [9], [10], an essential enzyme that relaxes DNA supercoils during DNA synthesis [11], [12].

ARF binding to topo I involves the C-terminal domain of ARF [13], which is not required for its p53-dependent activity, and is enhanced by protein kinase CK2 (“CK2”)-mediated hyperphosphorylation of topo I. We have shown that such aberrant topo I phosphorylation occurs on serine 506 (PS506) and is a cancer-related abnormality that is characteristic of cancer cells expressing elevated CK2 levels [9], [14]. We also found that PS506-expressing hyperphosphorylated topo I displays both increased DNA binding and increased DNA relaxation activity compared with the basal phosphorylated topo I expressed in normal cells and in cancer cells without elevated CK2 [15]. The elevated activity of hyperphosphorylated topo I also increases cellular sensitivity to the topo I-targeted drug, camptothecin [14], which uses topo I activity to generate lethal DNA double-strand breaks [16]. Thus, hyperphosphorylation of topo I is relevant to the cancer cell response to irinotecan and topotecan, two widely used camptothecin-derived chemotherapeutic drugs.

ARF–topo I complexes accumulate in cell lines with hyperphosphorylated topo I, and modulation of ARF levels in these cells affects topo I DNA relaxation activity and cellular sensitivity to camptothecin [9]. These findings suggest that the CK2-mediated hyperphosphorylation of topo I may drive the formation of ARF–topo I complexes to further increase topo I activity. ARF is rarely mutated in human cancer. However, overexpression of ARF occurs in 50% or more of a variety of cancers including lung, colon, and breast cancer, oral squamous cell carcinoma, and B-cell lymphoma [17]–[22]. PS506 expression may also be frequent in cancer, based on cell culture analyses showing it to be expressed in about two-thirds of cancer cell lines [15]. Because the ARF–topo I interaction may be relevant to a large fraction of cancers, arising as a consequence of chronic CK2 elevation and oncogene activation, we have further examined the molecular events controlling this interaction and the consequences for topo I function. Here, we show that ARF interacts with the core domain of topo I in a PS506-dependent manner to enhance the association of hyperphosphorylated topo I with DNA and chromatin. A pathophysiological role for hyperphosphorylated topo I is revealed by the finding that the combined expression of PS506 and ARF in cancer cells resulted in increased topo I-mediated DNA nicking and DNA double-strand break formation induced by elevated levels of reactive oxygen species. The results identify a novel activity of ARF, independent of its tumor suppressor activity, and raise the possibility that persistent overexpression of ARF could contribute to endogenous DNA damage in cancer cells by dysregulation of topo I.

## Materials and Methods

### Cell Lines

293T human embryonic kidney cells immortalized with T antigen, H358 human non-small cell lung cancer cells, and OVCAR-3 human ovarian cancer cells were purchased from the American Type Culture Collection (ATCC) and were cultured in Dulbecco’s Modified Eagles Medium (293T, OVCAR-3) or RPMI (H358) supplemented with 10% newborn calf serum and additives, as previously described [14].

### Antibodies

The primary antibodies used were: goat polyclonal anti-topo I and goat polyclonal anti-ARF C-terminus (Santa Cruz Biotechnology, Santa Cruz, CA); mouse monoclonal anti-phosphoserine, mouse monoclonal anti-FLAG, and rabbit polyclonal anti-FLAG (Sigma-Aldrich, St. Louis, MO); rabbit polyclonal anti-histone H2A.X and rabbit polyclonal anti-topo I (Bethyl Laboratories, Montgomery, TX); rabbit polyclonal anti–γ-H2A.X [ser139] (Novus Biologicals, Littleton, CO); mouse monoclonal anti-histone H3 (Millipore, Temecula, CA); and rabbit polyclonal anti-PS506 (pAb506-P; described in reference [23]). The secondary antibodies used were horseradish peroxidase (HRP)-conjugated goat anti-rabbit IgG, goat anti-mouse IgG-HRP, and donkey anti-goat IgG-HRP (all from Santa Cruz Biotechnology). For Westerns, primary and secondary antibodies were used at 1∶100 and 1∶1000 dilutions, respectively.

### Construction of Expression Vectors for Human topo I and topo I Deletion Mutants

The full-length human topo I sequence was PCR amplified from H358 cDNA and subcloned into the pCMV-Tag1 vector (Stratagene, San Diego, CA), which supplies an N-terminal FLAG sequence. The cloned insert was sequenced (Retrogen, San Diego, CA) and confirmed to be the human wild-type topoisomerase I coding sequence (NM_003286). The cloned full-length sequence was then used as a template to PCR amplify a series of C-terminal deletion mutants of varying lengths, the core region sequence (residues 216–636), and the region from residues 501−765 that includes part of the core domain, the linker, and the topo I C-terminus. The amplified deletion fragments were also inserted into the pCMV-Tag1 Vector (Stratagene) and all cloned inserts were then subcloned together with the FLAG sequence into the pTriEx™-2 Hygro vector (Novagen), which supplies a His-tag and T7 promoter for in vitro transcription/translation. Each cloned insert in pTriEx™-2 Hygro was sequenced to verify its identity. A sequence in which alanine replaced serine at position 506 was generated by site-direct mutagenesis as previously described [15]. The sequences of PCR primers are provided in Table S1.

### Recombinant topo I and ARF

With the exception of the His/FLAG-tagged human topo I expressed in cell lines, the recombinant topo I used in this study was baculovirus-expressed human topo I (R-topo I) purchased from TopoGEN (Port Orange, FL). The baculovirus R-topo I protein is basal phosphorylated. To generate hyperphosphorylated topo I for binding and activity assays, R-topo I was incubated twice, each for 30 min at 37°C, with 10 units of CK2 (Promega, Madison, WI) per microgram of R-topo I, as described in the manufacturer’s instructions. To generate unphosphorylated protein, basal phosphorylated R-topo I was dephosphorylated with alkaline phosphatase (Sigma) as previously described [9], [24]. The recombinant ARF (p14 Alternate Reading Frame protein) used here was a fusion protein of human ARF and thioredoxin expressed in bacteria as previously described [9].

### Evaluation of topo I–ARF Complex Formation

To evaluate intracellular complex formation between ARF and the products of the various topo I expression constructs, the vectors were transfected into 293T or H358 cells using TurboFect Transfection Reagent (Thermo Scientific, Waltham, MA) according to the manufacturer’s instructions. For each transfection, one 10 cm dish of nearly confluent cells was used (∼2×10^6^ cells). To increase cellular ARF levels in H358 cells, we treated them prior to transection for 4 h with 20 moi (multiplicity of infection) of Adp14, an adenoviral vector encoding human ARF, using procedures described previously [9]. Two days post-transfection, transduced cells were lysed by direct addition to the plates of high salt lysis buffer [400 mM NaCl, 50 mM Hepes pH 7.5, 1% Triton X-100, 10% glycerol, 5 mM MgSO_4_, 1 mM EDTA, and complete protease and phosphatase inhibitors (Roche, Indianapolis, IN)]. For H358 cells, the lysate was adjusted to 150 mM salt. Transduced gene products were immunoprecipitated overnight at 4°C with mouse anti-FLAG M2 and protein AG-agarose (Santa Cruz Biotechnology). Samples were subjected to Western analysis of FLAG (rabbit anti-FLAG), PS506, and ARF as described previously [9]. Western blots were developed using Pierce ECL Western blotting substrate (Thermo Scientific).

To evaluate complex formation between R-topo I and recombinant ARF in vitro, samples of 1 µg unphosphorylated, basal phosphorylated, or hyperphosphorylated R-topo I (prepared as described above) were incubated with 0.14 µg recombinant ARF fusion protein in lysis buffer (10 mM sodium phosphate pH 7, 150 mM NaCl, 0.1% SDS, 1% NP40, 1% sodium deoxycholate, 1 mM phenylmethylsulfonyl fluoride [PMSF], and complete protease inhibitors) as described previously [9]. Samples were immunoprecipitated overnight at 4°C with goat polyclonal anti-topo I and protein AG-agarose, and then subjected to SDS-PAGE and Western analysis of co-immunoprecipitated ARF.

### Western Analyses of Cell Lysates

Cells were lysed by the addition of high salt lysis buffer directly to the plates as described in the previous section, and the lysates were processed as previously described [14]. Samples of 75 µg cellular protein (except where noted differently) were resolved by SDS-PAGE, transferred to PVDF membranes, and immunostained as described above.

### Topo I–plasmid DNA Non-covalent Binding Assay

Binding assays with radiolabeled plasmid DNA and R-topo I were performed following our previously described method [15]. In brief, 0.03 pmol (600 ng, ∼9000 cpm) radiolabeled plasmid DNA (pCMV-FLAG, Sigma-Aldrich) and 0.3 pmol R-topo I (basal phosphorylated, hyperphosphorylated, or unphosphorylated prepared as described above) were incubated for various times with or without 0.3 pmol recombinant ARF in 50 µl low salt buffer (75 mM NaCl, 10 mM Tris, pH 7.5) at 4°C. These conditions allow topo I to associate with DNA but prevent catalytic DNA cleavage [25]. Non-covalent complexes were recovered by the addition of 250 µl low salt immunoprecipitation buffer (10 mM sodium phosphate pH 7, 75 mM NaCl, 0.1% SDS, 1% sodium deoxycholate, 0.5% NP40, and complete protease inhibitors), followed by goat anti-topo I and protein AG-agarose. Co-immunoprecipitated DNA was quantified by scintillation counting.

### Topo I DNA Nicking Assay

A radiolabeled suicide substrate was prepared using the sequence and method originally described in reference [25], and further detailed in reference [15]. The substrate is a 94 bp double-stranded hairpin structure containing a topo I binding and cleavage sequence 3 bases upstream of an engineered nick, in which the 5′-hydroxyl group required for resealing is replaced by a phosphate group. Therefore, once topo I has cleaved the suicide substrate, it remains trapped in a covalent complex with the DNA. Briefly, for the R-topo I assay in vitro, non-covalent complexes between the radiolabeled substrate and R-topo I were preformed by incubation in low salt buffer on ice, as described in the previous section. The temperature was then raised to 8°C for varying times to allow nicking and covalent complex formation. Reaction products were precipitated with K^+^SDS, a method that specifically recovers covalently linked protein–DNA complexes [15], [26] and is described further below (see “Recovery of cellular covalent cleavage complexes”). Precipitates were collected by centrifugation and DNA was quantified by scintillation counting.

### Topo I–plasmid DNA Relaxation Assay

The supercoiled plasmid DNA relaxation assay was performed using a Topo I Assay Kit (TopoGEN) and products were analyzed by agarose gel electrophoresis, as previously described [7].

### Chromatin Immunoprecipitation (ChIP) Assay

H358 cells were left untreated, or were treated with 10 µM of the CK2 inhibitor 4,5,6,7-tetrabromobenzotriazole (TBB) for 1 h as described in [14], with 20 moi Adp14 as described in [9], or with both TBB and Adp14. One 10 cm plate of cells at about 80% confluency was used for each condition. Two days later cells were crosslinked by addition of 1% formaldehyde for 10 min with shaking. Cells were washed twice with PBS and lysed by direct addition of 600 µl high salt lysis buffer to the plate. The lysate was sonicated to break the DNA into ∼1000 bp fragments and centrifuged. The supernatant was mixed with immunoprecipitation buffer (1% NP40, 20 mM Tris pH 8, 150 mM NaCl, 10% glycerol, 2 mM EDTA) and subjected to 3 cycles of immunoprecipitation with rabbit anti-histone H3 and protein AG-agarose, each for 2 h at 4°C. The immunoprecipitated fractions were pooled, resolved by SDS-PAGE, and subjected to Western analysis of topo I and histone H3.

### Recovery of Cellular Covalent Cleavage Complexes

OVCAR-3 cells were left untreated, or were treated with 20 moi of Adp14 as described in [9], 10 nM of the specific CK2 activator, 1-ethyl-4,5-dicarbamoyl imidazole [27] as described in [14], [15], or with both Adp14 and the CK2 activator. One 6 cm plate of cells (∼7×10^5^ cells) was used for each condition. To induce production of reactive oxygen species (ROS), cells were treated with 10 µM pyocyanin (Sigma) as described in [28]. ROS production was verified using an ROS detection kit (Enzo, Lake Placid, NY) according to the manufacturer’s instructions. Cells were incubated for a total of 48 h. After 29 h, cells were pulsed for 18 h with 1.2 µCi/plate of [^3^H]-thymidine (NEN Lifescience Products, Boston, MA) and then transferred to non-radioactive medium for the final 1 h before harvest. Cleavage complexes were isolated using a K^+^SDS method that specifically precipitates covalent topo I–DNA complexes, as originally described by Liu et al. [26] with some modifications [29], [30]. Briefly, after removing the medium, cells were lysed by direct addition to the plate of 200 µl of pre-warmed (65°C) lysis solution (1.25% SDS, 5 mM EDTA pH 8, 0.4 mg/ml salmon sperm DNA). Cell lysates were transferred to 1.5 ml microfuge tubes containing 50 µl of 325 mM KCl. After vigorous vortexing, the samples were cooled on ice for 10 min and centrifuged. The pellets were resuspended in 500 µl of wash solution (10 mM Tris-HCl pH 8, 100 mM KCl, 1 mM EDTA, 0.1 mg/ml salmon sperm DNA), warmed at 65°C for 10 min with occasional shaking, cooled on ice for 10 min, and re-centrifuged. The pellets were washed again and resuspended in 200 µl of water pre-warmed to 65°C. Radioactivity was determined by scintillation counting.

### Statistical Analyses

Statistical analyses were carried out using GraphPad® software (GraphPad Software, Inc., La Jolla, CA).

## Results

### Identification of the ARF-interacting Domain on topo I and the Role of PS506 in ARF–topo I Binding

**Figure 1:**
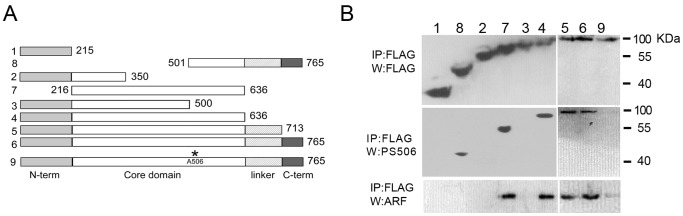
Identification of the ARF-interacting domain on topo I and the role of PS506 in ARF binding. (A) Scheme showing regions of the topo I protein encoded by the 9 expression constructs. The N-terminal domain, core domain, linker, and C-terminal domain are indicated. (B) Analysis of expression (top), PS506 content (middle), and ARF binding (bottom) of the products of constructs 1–9 expressed in human H358 lung cancer cells. Cells were treated with Adp14 (20 moi) to elevate cellular ARF levels. Lysates were collected 2 days after treatment and subjected to mouse anti-FLAG immunoprecipitation (IP) followed by Western (W) analysis with antibodies specific for FLAG (rabbit anti-FLAG), PS506, or ARF, as indicated.

Topo I consists of 4 domains: a poorly conserved N-terminal domain, a highly conserved DNA-binding core domain containing most of the catalytic residues, a poorly conserved linker domain, and a conserved C-terminal domain containing the catalytic residue tyrosine 723 (see review [31] and references therein). To map the ARF binding domain of topo I we generated His/FLAG-tagged expression vectors for the full-length wild-type topo I, a full-length site-specific topo I mutant in which alanine replaces serine at position 506, and a series of 7 deletion mutants (Figure 1A). The 9 constructs were expressed in H358 human lung cancer cells, which we have previously shown to have elevated levels of CK2 and ARF and to express the PS506-hyperphosphorylated form of topo I [15]. Cells were also treated with the human ARF adenoviral vector Adp14 to further increase ARF expression. Two days after transfection, the topo I gene products were immunoprecipitated from cell lysates with mouse anti-FLAG IgG, and probed by Western analysis for the topo I proteins, PS506, and co-immunoprecipitated ARF.

As shown in Figure 1B, the 9 topo I constructs were all efficiently expressed in H358 cells (anti-FLAG Western). The presence of the PS506 epitope was probed with pAb506-P, a rabbit polyclonal IgG raised to a PS506-containing topo I peptide, which we previously showed recognizes PS506-containing topo I, but not the unphosphorylated or basal phosphorylated forms of topo I [15]. As expected, we observed immunoreactivity of pAb506-P with the products of constructs 4−8, which all include serine 506, but not with constructs 1−3, which lack serine 506. Similarly, the PS506 epitope was not present in construct 9 containing the S506A substitution, consistent with our earlier report [15].

ARF binding to the transduced gene products was evaluated by Western analysis of ARF in the topo I immunoprecipitates. We found that the products of constructs 1–3 could not form complexes with ARF, indicating that the first 500 residues of topo I are insufficient for ARF binding (Figure 1B) but the addition of residues 500 to 636 (construct 4) enabled binding to ARF. Notably, the product of construct 4 bound ARF as efficiently as the products of constructs 5 and 6, which contain the linker and C-terminal regions of topo I, indicating that these regions are unlikely to contribute further to ARF binding. The core region from residue 216 to 636 (construct 7), devoid of N-terminal sequences, linker, and C-terminal sequences was able to bind ARF, indicating that the N-terminal region is also dispensable for binding. However, the region from residue 500 to 636 (in construct 8) in the absence of the remainder of the core region, did not bind ARF, indicating that a larger region of the core domain was necessary for binding, possibly to retain the proper folding. We also expressed these constructs in 293T cells and evaluated binding of the products to a bacterially expressed recombinant human ARF fusion protein, and obtained similar results (Figure S1). Importantly, we found that efficient binding of ARF by full-length topo I was PS506-dependent. Thus, ARF binding by the S506A gene product (construct 9), which cannot be phosphorylated at residue 506 and does not express PS506, is greatly impaired compared with binding by the PS506-positive full-length wild-type topo I (construct 6; Figure 1B).

### Effect of PS506 and ARF on the DNA Binding and Relaxation Activity of topo I

**Figure 2:**
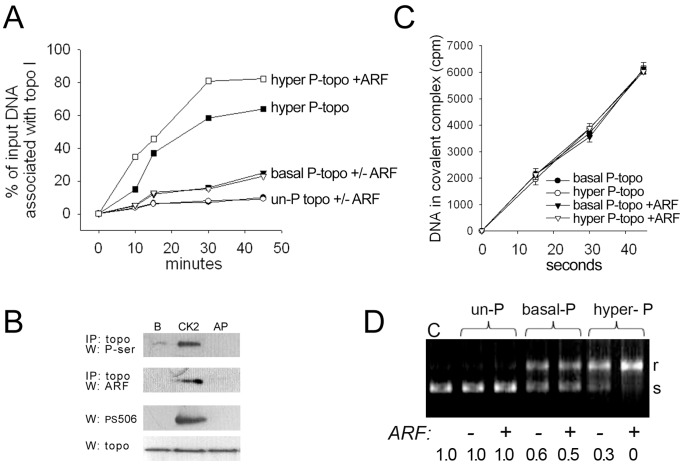
Effects of topo I phosphorylation status and ARF on topo I DNA association and relaxation activity. (A) Time course of non-covalent association [low salt (75 mM NaCl) at 4°C] of various R-topo I forms (0.3 pmol) with 0.03 pmol [^3^H]-labeled plasmid DNA in the presence or absence of 0.3 pmol bacterially produced human ARF fusion protein: hyperphosphorylated topo I minus ARF (▪) or plus ARF (□); basal phosphorylated topo I minus ARF (▾) or plus ARF (▿); and unphosphorylated (AP-treated) topo I minus ARF (•) or plus ARF (○). (B) Row 1: Topo I immunoprecipitation (IP) with basal phosphorylated (lane “B”), hyperphosphorylated (lane “CK2”), and unphosphorylated (lane “AP”) R-topo I (1 µg) followed by phosphoserine Western; **row 2:** topo I IP of basal phosphorylated, hyperphosphorylated, or unphosphorylated R-topo I (1 µg) incubated with 0.14 µg ARF, followed by ARF Western; rows 3 and 4: Western analyses of PS506 and total topo I, respectively, with the same basal phosphorylated, hyperphosphorylated, and unphosphorylated R-topo I samples as in rows 1 and 2 (0.3 µg per lane) (**C**) Rate of topo I-catalyzed nicking of a radiolabeled suicide substrate that traps topo I and DNA in a covalent complex. Non-covalent complexes of DNA with basal or hyperphosphorylated R-topo I were preformed by incubation in low salt at 4°C in the presence or absence of recombinant ARF, then the temperature was raised to 8°C for the indicated times. Covalently linked DNA-topo I complexes were recovered by precipitation with K^+^SDS and quantified by scintillation counting. (D) Topo I-mediated plasmid relaxation assay performed with unphosphorylated, basal phosphorylated, and hyperphosphorylated R- topo I, followed by agarose gel electrophoresis to separate substrate and products; s = supercoiled, r = relaxed plasmid DNA.

The PS506 epitope can be generated in vitro by CK2 treatment of basal phosphorylated, baculovirus-expressed recombinant topo I (R-topo I) (Materials and Methods and reference [15]). We have previously shown that the basal and hyperphosphorylated forms of R-topo I have similar catalytic nicking rates on a synthetic DNA substrate, but the hyperphosphorylated form shows higher non-covalent association with plasmid DNA [15]. Here, we used similar methods to determine how ARF affects the non-covalent DNA association and catalytic rate of the basal and hyperphosphorylated forms of R-topo I. For comparison, we also tested an unphosphorylated R-topo I formed by alkaline phosphatase treatment of basal phosphorylated R-topo I, as described in Materials and Methods. The phosphoserine and PS506 status of each of the R-topo I forms used in the DNA binding and activity assays was confirmed by Western analyses (Figure 2B).

Non-covalent complex formation between the R-topo I forms and radiolabeled plasmid DNA was performed under low salt (75 mM NaCl), low temperature (4°C) conditions, which allow topo I–DNA association but prevent catalytic nicking and enzyme dissociation [15], [25]. Complexes were recovered by topo I immunoprecipitation, and co-precipitated labeled DNA was quantified by scintillation counting. As shown in Figure 2A, the fraction of input DNA associated with topo I was dependent on the topo I phosphorylation status. At 30 min, less that 10% of input DNA was associated with the unphosphorylated R-topo I, but this increased to ∼20% for the basal phosphorylated form, and to ∼60% for the hyperphosphorylated form. The low level of co-immunoprecipitated DNA observed for the unphosphorylated form of R-topo I under these conditions is consistent with other reports showing that a basal level of topo I serine phosphorylation is critical for catalytic activity [32]–[34].

The addition of a recombinant thioredoxin-human ARF fusion protein at a 1∶1 molar ratio enhanced the non-covalent association of hyperphosphorylated R-topo I to radiolabeled plasmid DNA, resulting in the co-immunoprecipitation of about 80% of the DNA (Figure 2A). In contrast, ARF addition had virtually no effect on the DNA association of the basal phosphorylated or unphosphorylated forms of R-topo I, consistent with the observation that hyperphosphorylated R-topo I bound strongly to ARF, whereas neither the basal nor unphosphorylated forms displayed detectable ARF binding (Figure 2B). Increasing the molar ratio of ARF to hyperphosphorylated topo I did not further increase the DNA association (data not shown). Collectively, the findings that ARF binds to and enhances the DNA association of only the hyperphosphorylated form of R-topo I, and that DNA binding is maximal when the proteins are present at an equimolar ratio, indicate that ARF increases the DNA association of hyperphosphorylated topo I through the formation of a heterodimeric ARF–topo I complex.

We next examined the effect of ARF on the catalytic rate of basal phosphorylated and hyperphosphorylated R-topo I. The first step in topo I-mediated relaxation of DNA supercoils is the generation of a single-strand nick that allows the uncleaved strand to swivel. Nicking generates a transient intermediate termed a “cleavage complex” in which tyrosine 723 in the active site of topo I becomes covalently bound to the 3′-end of the nick (reviewed in [31]). Following DNA unwinding, the nick is resealed by topo I and the covalent topo I–DNA linkage is broken. To assay for topo I-mediated single-strand nicks, we captured cleavage complexes using a radiolabeled synthetic DNA substrate originally described by Soe et al [35]. The substrate provides a preferred topo I binding site but is designed in such a way that it cannot reseal, trapping topo I in a covalent linkage with DNA. We have previously used this substrate to show that CK2 treatment of basal phosphorylated R-topo I does not alter the rate of single-strand nicking [15]. To determine how ARF affected the rate of topo I-mediated single-strand nicking, we first established preformed non-covalent complexes between the radiolabeled DNA substrate and basal phosphorylated or hyperphosphorylated R-topo I under low salt conditions at 4°C, as in Figure 2A. ARF was then added to an equimolar ratio with topo I and the catalytic reaction was initiated by raising the temperature to 8°C. The reactions were stopped by the addition of a K^+^SDS solution to precipitate proteins covalently bound to DNA, and DNA was quantified by scintillation counting. As expected, no covalent complexes were recovered at time zero at 4°C, confirming that catalysis was not occurring under these conditions (Figure 2C). In contrast, covalent complexes were formed in a time-dependent manner at 8°C. The reaction kinetics were unaffected by the topo I phosphorylation state or the presence of ARF, indicating that topo I hyperphosphorylation and topo I–ARF complex formation are important to DNA binding, but do not affect the catalytic rate of topo I once bound to DNA.

Topo I relaxation activity is influenced by both the extent of DNA binding and the catalytic rate. We examined the combined effects of topo I hyperphosphorylation and complex formation with ARF on DNA relaxation activity by incubating supercoiled plasmid DNA with unphosphorylated, basal phosphorylated, or hyperphosphorylated R-topo I in the presence or absence of ARF (Figure 2D). To highlight the effects of ARF, we performed the experiment under conditions in which conversion of the supercoiled plasmid to the relaxed form was suboptimal for all forms of topo I. The conversion of the supercoiled plasmid to the relaxed form was revealed by the differential electrophoretic mobility of these two forms on agarose gel electrophoresis, and the intensities of the supercoiled bands were quantified digitally. We found that unphosphorylated topo I was inactive in this assay in both the presence and absence of ARF, consistent with the poor binding of this form of topo I to DNA and with the failure of ARF to influence its DNA binding (Figure 2A). Basal phosphorylated R-topo I converted about 40% of the supercoiled plasmid to the relaxed form, and this was enhanced marginally (to 50%) by the presence of ARF. This result is consistent with the positive effect of basal phosphorylation on topo I−DNA binding, while having a negligible effect on topo I−ARF binding (Figures 2A,B, respectively). Hyperphosphorylated R-topo I converted ∼70% of supercoiled DNA to the relaxed form and this was increased to virtually 100% by the addition of ARF, reflecting the enhanced binding of hyperphosphorylated topo I to both DNA and ARF (Figures 2A,B, respectively). Taken together, these results support a model in which the effects of elevated levels of cellular ARF and CK2 converge on topo I to increase topo I−DNA binding and DNA relaxation.

### Chromatin Association of topo I

**Figure 3:**
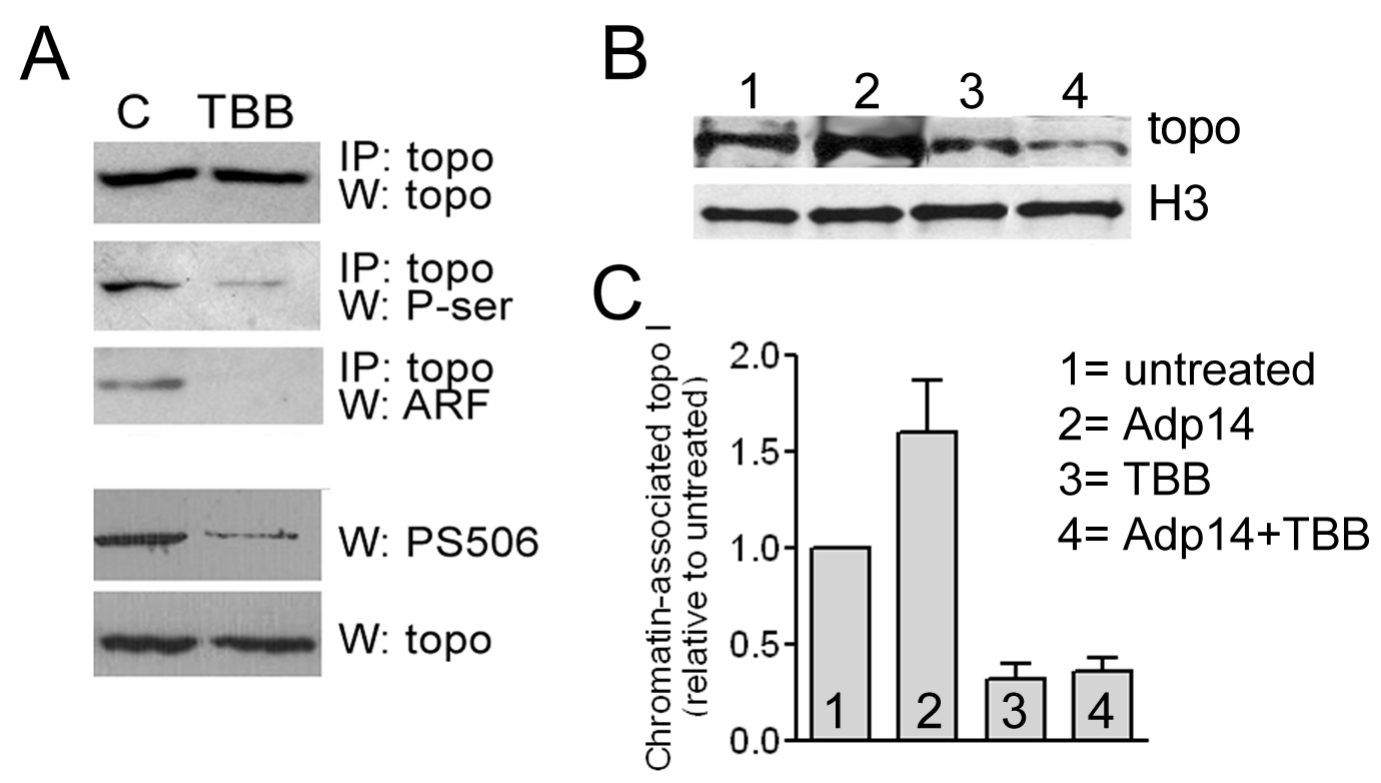
Chromatin association of topo I. (A) Rows 1–3: Topo I IP followed by Western analyses of total topo I, phosphoserine, and ARF was performed before (lane “C”) or 2 days after (lane “TBB”) treatment of H358 cells with TBB (10 µM for 1 h); rows 4 and 5: Western analyses of PS506 and total topo I in the same starting samples as in rows 1–3. Quantification of band densities indicated that TBB treatment reduced both P-ser and PS506 reactivity by ∼80%. (B) Histone H3 chromatin immunoprecipitation (ChIP) of untreated H358 cells (lane 1) or 2 days after treatment with 20 moi Adp14 (lane 2), TBB (10 µM, 1 h; lane 3), or both Adp14 and TBB (lane 4), followed by Western analyses of histone H3 and topo I. (C) The results of four independent ChIP analyses performed as in (B); bars represent the mean and standard deviation of chromatin-associated topo I levels in the treated cells relative to the untreated cells, quantified digitally from band intensities.

To determine how PS506 expression and ARF complex formation affects topo I association with chromatin in cells, we performed chromatin immunoprecipitation (ChIP) analyses in H358 cells before and after experimental modulation of endogenous CK2 and ARF levels. H358 cells express ARF, but the levels can be increased about 3-fold at 2 days following exposure to 20 moi of Adp14 [9]. Virtually all of the topo I in these cells is in the hyperphosphorylated form [15]. Treatment of H358 cells with the CK2 inhibitor TBB reduced topo I serine phosphorylation and PS506 expression by about 70% and 80%, respectively (based on digital quantification of band intensities in Figure 3A) and abolished topo I–ARF binding (Figure 3A). For ChIP assays, H358 cells were left untreated, or were treated with TBB for 1 h, Adp14, or both TBB and Adp14, and then incubated for an additional 2 days. None of the treatments affected cell viability, as determined by visual examination and trypan blue exclusion (data not shown). Chromatin from formaldehyde-fixed, sonicated cells was then immunoprecipitated with anti-histone H3 IgG, and the material was subjected to Western analysis to detect topo I and histone H3. As shown in Figure 3B, equivalent amounts of histone H3 were recovered from all samples, but the levels of chromatin-associated topo I were increased in the Adp14-treated cells (lane 2) and decreased in TBB-treated cells (lane 3), compared with untreated cells (lane 1); Adp14 treatment had no effect on chromatin-bound topo I levels in TBB-treated cells (lane 4). These results were confirmed in three additional independent experiments. For the four experiments, the band intensities were quantified and averaged to determine the average amount of topo I bound to chromatin for each treatment condition relative to the untreated cells (Figure 3C). The results showed that the level of chromatin-associated topo I in cells treated with Adp14 alone was increased ∼1.5–2-fold compared with untreated cells, indicating that ARF enhanced the association of hyperphosphorylated, PS506-containing topo I to chromatin. The level of chromatin-associated topo I in cells treated with TBB was ∼50% of that in untreated cells, indicating that loss of PS506 expression correlated with reduced topo I−chromatin association. Adp14 treatment did not significantly enhance topo I−chromatin association from TBB-treated cells, indicating that ARF does not promote the chromatin association of basal or unphosphorylated topo I, consistent with its inability to bind these forms of topo I. The observed differences between control and Adp14 treated cells, between control and TBB-treated cells, and between control and Adp14+TBB treated cells were statistically significant (p<0.05 in each case, based on a ratio t-test), but the difference between TBB treatment and Adp14+TBB treatment was not statistically significant. Thus, the effect of ARF on the association of hyperphosphorylated topo I with chromatin in cells parallels its effects on topo I–plasmid DNA binding in vitro.

### Topo I-mediated DNA Damage

Because the physiological role of topo I is to induce single-strand DNA break formation during DNA replication and because this can become a double-strand DNA break under certain conditions, we asked whether the increased chromatin association of the hyperphosphorylated, ARF-bound form of topo I would enhance DNA single- or double-strand break formation in cells. As mentioned above, a single-strand break occurs when topo I initiates DNA relaxation by cleaving one strand of the double helix to allow for swiveling of the uncleaved strand. Single-strand cleavage is accompanied by the covalent linkage of topo I to the 3′-end of the DNA to form the transient cleavage complex intermediate. A DNA double-strand break occurs when the normally transient cleavage complex is aberrantly stabilized, causing the single-strand break to persist and become a double-strand break upon encounter with an advancing replication fork. Topo I-targeted drugs such as camptothecin and related chemotherapeutic agents are thought to kill cells by stabilizing the cleavage complex and promoting DNA double-strand break formation during DNA synthesis [16]. Oxidized DNA bases such as 7,8-dihydro-8-oxoguanine and 5-hydroxycytosine that result from oxidative stress can also interfere with the completion of the topo I reaction [36], potentially causing the single-strand DNA break to persist and become a DNA double-strand break during DNA synthesis. This is of particular relevance to the function of topo I in cancer cells because increased formation of reactive oxygen species (ROS) often accompanies oncogene activation and is thus an endogenous source of oxidative stress in cancer cells [37], [38].

**Figure 4:**
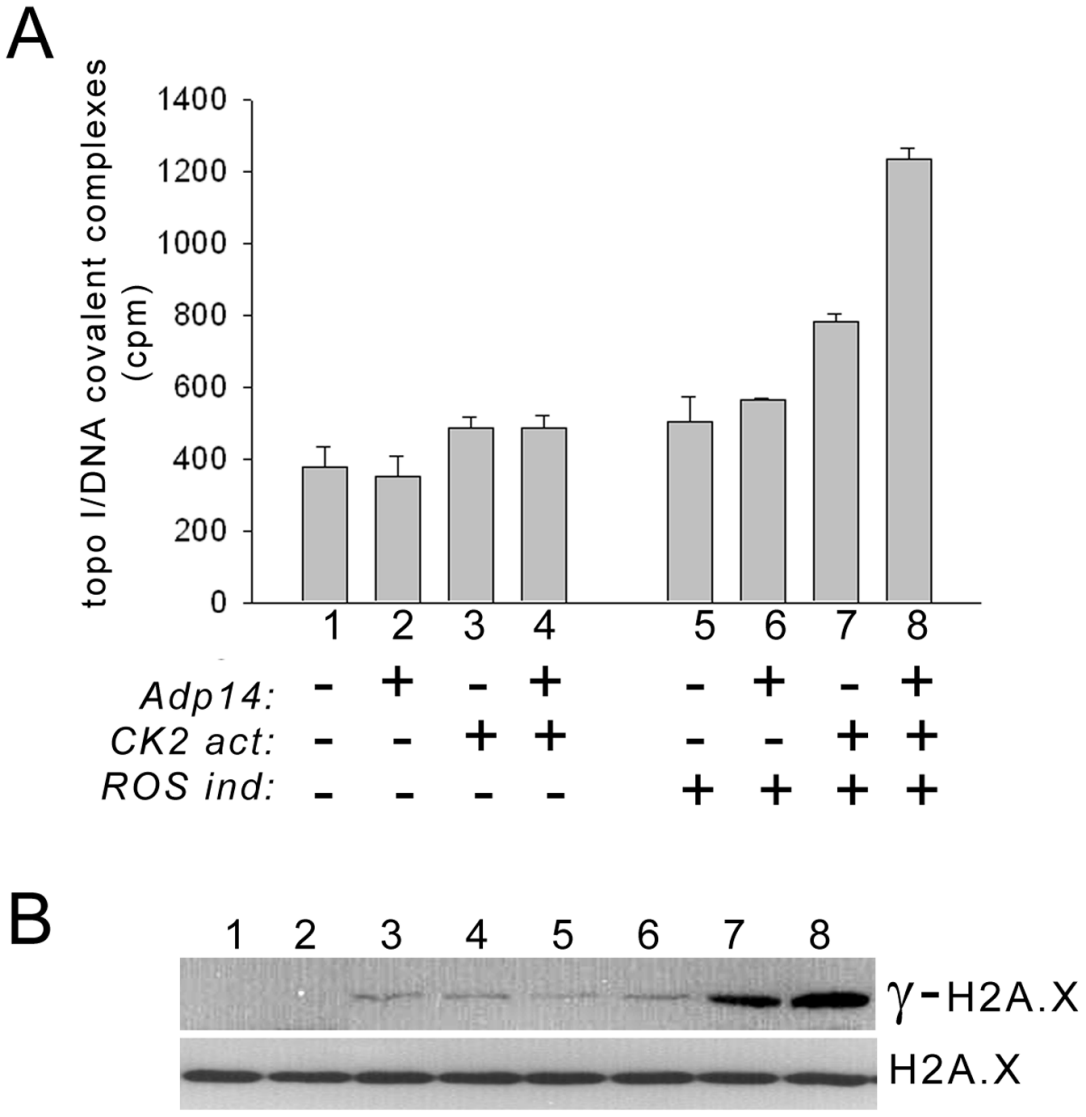
Topo I cleavage complex formation and induction of DNA double-strand breaks. (A) Cleavage complex formation in untreated OVCAR-3 cells (bars 1,5), or 2 days after treatment with 20 moi Adp14 (bars 2,6), 10 nM of the CK2 activator 1-ethyl, 4,5 dicarbamoyl imidazole (bars 3,7), or both Adp14 and the CK2 activator (bars 4,8). Samples 5–8 were also treated with 10 µM of the ROS inducer pyocyanin. Cells were pulsed with [^3^H]-thymidine to label DNA, cleavage complexes were captured by K^+^SDS precipitation, and DNA was quantified by scintillation counting. (B) Western analysis of γ-H2A.X, an indicator of DNA double-strand break formation, in lysates of H358 cells subjected to the same treatments 1–8 as in part (A). Total H2A.X levels are shown as a control.

To determine how modulation of ARF and ROS levels and topo I phosphorylation affect the formation of topo I cleavage complexes, we examined OVCAR-3 cells, which have low levels of CK2 and therefore express basal phosphorylated topo I that lacks PS506 [14], [15]. To increase CK2 levels and induce PS506 expression, we treated cells with the CK2 activator, 1-ethyl, 4,5 dicarbamoyl imidazole, which we previously showed increases CK2 activity 4-fold and induces expression of PS506 [15]. Although OVCAR-3 cells express ARF, ARF–topo I complexes were undetectable in untreated OVCAR-3 cells, but were induced by CK2 activator treatment and were further elevated by treatment with 20 moi of Adp14, which increases ARF levels about 3-fold by 48 h (Figure S2). To increase ROS levels, cells were treated with pyocyanin, a redox-active bacterial protein [28]. Two days after cells were treated with varying combinations of CK2 activator, Adp14, and pyocyanin, cleavage complexes were recovered from cell lysates using the K^+^SDS precipitation procedure described for Figure 2. The recovery of cleavage complexes was similar for untreated and Adp14-treated cells (Figure 4A, bars 1,2) and was marginally increased by the addition of the CK2 activator in both cases (Figure 4A, bars 3,4). ROS induction, either alone or in the presence of Adp14, also marginally increased the recovery of cleavage complexes (Figure 4A, bars 5,6). However, cleavage complex recovery was increased ∼2-fold when cells were treated with the combination of the ROS inducer and CK2 activator (Figure 4A, compare bars 7 and 1), and this was further increased to ∼3 times the level in untreated cells by the addition of Adp14 (Figure 4A, bar 8 versus bar 1). Thus, topo I-mediated single-strand DNA nicking and cleavage complex stabilization is enhanced by the combined effects of ROS elevation, topo I hyperphosphorylation, and ARF overexpression.

Because aberrant stabilization of cleavage complexes can precede the formation of DNA double-strand breaks, we evaluated DNA double-strand break formation in treated OVCAR-3 cells by detection of the histone variant, γ-H2A.X, a phosphorylated form of histone H2A.X that serves as a sensitive indicator of DNA double-strand break formation [39]. We found that levels of γ-H2A.X were undetectable in untreated or Adp14-treated OVCAR-3 cells (Figure 4B, lanes 1, 2) and were increased slightly in response to CK2 activation or ROS induction in the presence or absence of ARF overexpression (Figure 4B, lanes 3–6). However, ROS induction combined with CK2 activation markedly increased the accumulation of γ-H2A.X (Figure 4B, lane 7), and this was further increased by Adp14 treatment (Figure 4B, lane 8). Notably, these data paralleled the treatment effects on cleavage complex formation (Figure 4A). Importantly, cell viability was not affected by CK2 activator treatment, Adp14 treatment, or ROS induction, or by the combination of these treatments (data not shown), and γ-H2A.X accumulation was only about 2% of the level observed 2 days after a cytotoxic treatment with camptothecin (see Figure S3). Taken together, these results are consistent with a model in which the increased occupation of chromatin with hyperphosphorylated, ARF-associated topo I, particularly in the presence of elevated ROS production, promotes aberrant nicking of DNA by topo I that could lead to increased generation of double-strand DNA breaks.

## Discussion

The results of this study confirm and extend studies by ourselves and others that an interaction between topo I and the ARF C-terminus stimulates topo I-mediated DNA relaxation [10], [13] in a topo I phosphorylation-dependent manner [9], [14]. The interaction defines a novel role for ARF in enhancing topo I-generated DNA strand breaks that is completely distinct from its p53-dependent tumor suppressor activity. We show here that the interaction involves the topo I core domain and is strongly enhanced by phosphorylation on serine 506 (PS506), located within this domain. As we have previously reported, the PS506 epitope is a feature of an aberrant, hyperphosphorylated form of topo I with enhanced DNA binding and DNA relaxation activities present in cancer cell lines expressing elevated CK2 levels, but not in cell lines derived from normal tissues or in cancer cell lines with low levels of CK2 [14], [15]. Here we show that the enhanced DNA binding and DNA relaxation activities of hyperphosphorylated R-topo I in vitro are further increased by ARF, and that In cancer cells, hyperphosphorylation of topo I increases its association with chromatin, and this is further increased by ARF expression. This mechanism is likely to account for the ability of ARF to increase CPT cytotoxicity in cancer cells with hyperphosphorylated topo I as we previously observed [9].

Based on X-ray crystallographic studies of topo I associated with DNA, serine 506 occupies an internal location near DNA binding residues but not in contact with DNA itself [40]. The internal location of serine 506 in the DNA-bound topo I would appear to render it inaccessible to CK2 or ARF. However, in the DNA-unbound enzyme there may be enough structural flexibility to exposure this region. It has been proposed that for DNA binding to occur, the enzyme must initially exist in a more open conformation [40]–[42]. This may offer an opportunity for phosphorylation of serine 506, and this phosphorylation could favor a conformation poised for interaction with DNA. Interaction with ARF may also be favored with a more open topo I. As a positively-charged protein with nucleic acid-binding properties [43], ARF may promote the association of topo I with DNA or chromatin, but additional studies will be needed to determine if ARF remains associated with DNA-bound topo I. Because we observe that the basal and hyperphosphorylated forms of topo I with or without ARF display the same catalytic rate, we conclude that once the enzyme has established contacts with DNA, neither the presence of PS506 nor the addition of ARF affects the internal molecular dynamics involved in catalysis.

Under normal physiological conditions, CK2 is expressed at low and relatively unchanging levels [44], and ARF is usually undetectable. CK2 expression is increased in many cancers [45]–[50] and is associated with increased dysplasia, tumor aggressiveness, and poor prognosis [46], [48], [51]–[54], suggesting that CK2 may contribute to malignant progression. ARF levels increase as one of the earliest responses to oncogene-driven cellular hyperproliferaton [1]–[5]. ARF interacts via its N-terminal domain with mdm2, the key negative regulator of p53, and thus promotes p53 accumulation and p53-mediated cell growth arrest, senescence, or apoptosis [8]. ARF has also been suggested to play a p53-independent, mdm2-independent role in tumor suppression, although this function remains poorly understood (reviewed in [55]). Importantly, ARF has been shown to associate via its N-terminus with the nucleolar protein nucleophosmin, which stabilizes ARF and promotes its accumulation in the nucleolus [56]. ARF is then available to interact through its C-terminus with topo I, which is also a predominantly nucleolar protein. The ARF–topo I complex may therefore arise as a consequence of oncogene activation and elevated CK2 levels, and the effects of this complex may become particularly significant in cells where p53-mediated apoptosis is disabled.

ARF–topo I complexes are not detected in normal cells or in cancer cells that lack hyperphosphorylated topo I [14]. However, in cancer cells that accumulate ARF and express PS506, these complexes are readily detectable and sequester virtually all of the cellular ARF ([9] and our unpublished data). As we have previously reported, cancer cell lines with hyperphosphorylated topo I tend to be more sensitive to camptothecin-induced DNA damage [14], and this sensitivity can be modulated by manipulating ARF levels [9]. It seems likely that these effects could be attributed to the increased chromatin association of hyperphosphorylated, ARF-bound topo I, which would be highly relevant to the clinical use of camptothecin-based therapeutics. Although multiple factors influence the clinical response to such drugs, the expression of PS506 or the formation of ARF–topo I complexes could be important indicators of dysregulated topo I function, and serve as minimal biomarkers of potential chemosensitivity.

In addition to being a target for chemotherapy, dysregulated topo I could play a role in the underlying mechanism of malignancy by contributing to genome instability. We observed that formation of topo I cleavage complexes was increased by topo I hyperphosphorylation and ARF binding and correlated with the increased formation of DNA double-strand breaks. Such DNA breaks are common intermediates during the formation of genomic abnormalities accompanying malignancy, including DNA rearrangements, deletions, and amplifications. At the cleavage complex stage, topo I can carry out religation with a non-homologous DNA strand, thereby promoting recombination [57]–[59]. Yeast overexpressing topo I have a 6–12-fold increase in illegitimate recombination (non-homologous end-joining) [60] and vaccinia topo I promotes illegitimate recombination in *E. coli* [61]. In human cancer cells, DNA lesions resulting from ROS-induced DNA damage might also stimulate topo I recombinogenic activity by impeding intrachain rejoining following topo I-mediated single-strand breakage, which would leave the free DNA end available for interchain recombination with a non-homologous DNA. A recent report linking topo I to DNA breakage at chromosomal common fragile sites provides additional evidence for a role for topo I in generating genome instability [62]. Chromosomal common fragile sites are normally stable but become hotspots for chromosomal rearrangements in cancer cells [63]. We found that cells with hyperphosphorylated topo I, overexpressed ARF, and elevated ROS levels had increased levels of chromatin-associated topo I, topo I cleavage complexes, and DNA double-strand breaks, suggesting that these cellular features provide conditions favorable for the generation of topo I-mediated DNA breakage and rejoining, which might contribute to malignant progression.

**Figure 5:**
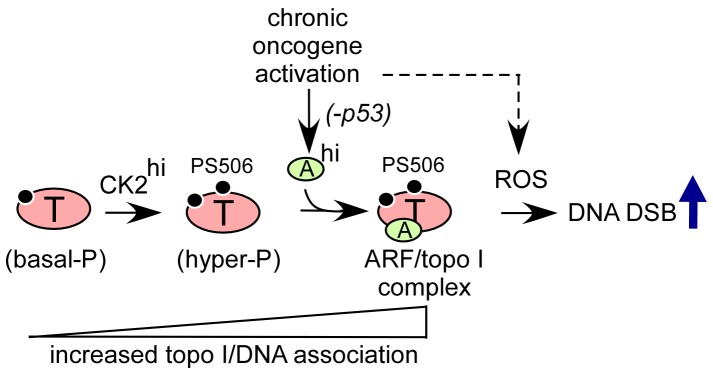
Model summarizing how oncogene-induced ARF expression and CK2-mediated topo I hyperphosphorylation can converge to enhance topo I–DNA association and topo I-facilitated DNA damage. Cancer cells with elevated CK2 levels (CK2^hi^) accumulate a PS506-hyperphosphorylated form of topo I with increased DNA binding properties. Chronic oncogene activation in the absence of wild-type p53 leads to sustained elevation of ARF (ARF^hi^), which is unable to promote p53-mediated apoptosis but is available to bind to PS506-hyperphosphorylated topo I, further promoting the association of topo I with DNA. The enhanced binding increases the potential for topo I-facilitated DNA double-strand break (DSB) formation in the presence of elevated levels of reactive oxygen species (ROS) that accompany oncogene activation.

Taken together, the data suggest a model shown in Figure 5. A basal level of topo I phosphorylation, involving sites distinct from PS506, is a minimal requirement for topo I association with DNA. This is consistent with reports in the literature that topo I serine phosphorylation is required for enzymatic activity [32]–[34]. These basal phosphorylation sites may include serine residues 112 and 394, which were previously reported to be targeted by Cdk1, and serines 21 and 10, which were previously reported to be targeted by PKC and CK2, respectively [64]. The DNA and chromatin association of basal phosphorylated topo I is increased by CK2-mediated phosphorylation of serine 506, which produces an aberrantly hyperphosphorylated form of topo I in cancer cells with elevated CK2 levels. ARF binding to the hyperphosphorylated topo I core domain further increases topo I association with DNA. These events increase the likelihood that an aberrantly stabilized cleavage complex will form, particularly in cells with elevated levels of ROS, which could generate double-strand breaks during DNA synthesis and have potential consequences for genome integrity. Further studies will help to elucidate the role of the ARF–topo I complex in malignant progression and to determine how this complex might be exploited therapeutically and diagnostically in cancer.

## Supporting Information

Figure S1 **Analysis of ARF binding to the products of constructs 1–6 (see** **Figure 1A** **) expressed in 293T immortalized human embryonic kidney cells.** Two days after transfection (one 10 cm dish of cells per transfection), transduced gene products were recovered by lysing cells in high salt buffer (see Materials and Methods) followed by selection on cobalt-agarose (Thermo Scientific). The gene products were eluted in 60 µl of 1 M imidazole elution buffer (Thermo Scientific) and added to 1 ml of immunoprecipitation buffer (20 mM Tris pH 8, 150 mM NaCl, 10% glycerol, 1% NP40, 2 mM EDTA, and complete protease inhibitors. Products were mixed with 3 µg bacterially expressed recombinant human ARF and subjected to FLAG IP followed by Western analysis of FLAG or ARF, as described for Figure 1B.
(TIF)

Figure S2 **ARF–topo I complexes in OVCAR-3 cells in the presence or absence of CK2 activation and ARF overexpression.** OVCAR-3 cells were treated with 20 moi of Adp14 (4 hr) with or without CK2 activator treatment (10 nM, duration of experiment). Top row: 2 days later, cells were analyzed by ARF Western to determine ARF levels. Digital quantification of bands relative to untreated is indicated in italics below lanes. Middle and Bottom rows: topo I IP followed by ARF and topo I in same samples to detect the formation of ARF–topo I complexes.
(TIF)

Figure S3 **Comparison of DNA damage in OVCAR-3 cells after various treatments.** Lane 1: Western analysis of γ-H2A.X and H2A.X (control) in OVCAR-3 cells, 2 days after treatment with 10 nM CK2 activator (duration of experiment) plus 80 nM camptothecin (first 18 h), a cytotoxic treatment that reduces cell viability to 20% of that observed with untreated cells [15] (lane 1); Lane 2: Western analysis of γ-H2A.X and H2A.X (control) in OVCAR-3 cells, 2 days after a combination treatment with 20 moi Adp14 (4 hours), 10 nM CK2 activator (duration of experiment) and 10 µM pyocyanin (duration of experiment). Treatment conditions same as for lane 8 of Figure 4B. Each lane represents 20 µg cellular protein.
(TIF)

Table S1 **Primers used for generating topo I deletion fragments.**
(DOCX)

## References

[1] Bates S, Phillips AC, Clark PA, Stott F, Peters G, et al (1998) p14ARF links the tumour suppressors RB and p53. Nature 395: 124–125. 9744267 10.1038/25867

[2] de Stanchina E, McCurrach ME, Zindy F, Shieh SY, Ferbeyre G, et al (1998) E1A signaling to p53 involves the p19(ARF) tumor suppressor. Genes Dev 12: 2434–2442. 9694807 10.1101/gad.12.15.2434 PMC317046

[3] Palmero I, Pantoja C, Serrano M (1998) p19ARF links the tumour suppressor p53 to Ras. Nature 395: 125–126. 9744268 10.1038/25870

[4] Radfar A, Unnikrishnan I, Lee HW, DePinho RA, Rosenberg N (1998) p19(Arf) induces p53-dependent apoptosis during abelson virus-mediated pre-B cell transformation. Proc Natl Acad Sci U S A 95: 13194–13199. 9789064 10.1073/pnas.95.22.13194 PMC23757

[5] Zindy F, Eischen CM, Randle DH, Kamijo T, Cleveland JL, et al (1998) Myc signaling via the ARF tumor suppressor regulates p53-dependent apoptosis and immortalization. Genes Dev 12: 2424–2433. 9694806 10.1101/gad.12.15.2424 PMC317045

[6] Zindy F, Williams RT, Baudino TA, Rehg JE, Skapek SX, et al (2003) Arf tumor suppressor promoter monitors latent oncogenic signals in vivo. Proc Natl Acad Sci U S A 100: 15930–15935. 14665695 10.1073/pnas.2536808100 PMC307670

[7] Honda R, Yasuda H (1999) Association of p19(ARF) with Mdm2 inhibits ubiquitin ligase activity of Mdm2 for tumor suppressor p53. Embo J 18: 22–27. 9878046 10.1093/emboj/18.1.22 PMC1171098

[8] Midgley CA, Desterro JM, Saville MK, Howard S, Sparks A, et al (2000) An N-terminal p14ARF peptide blocks Mdm2-dependent ubiquitination in vitro and can activate p53 in vivo. Oncogene 19: 2312–2323. 10822382 10.1038/sj.onc.1203593

[9] Bandyopadhyay K, Lee C, Haghighi A, Baneres JL, Parello J, et al (2007) Serine phosphorylation-dependent coregulation of topoisomerase I by the p14ARF tumor suppressor. Biochemistry 46: 14325–14334. 18004878 10.1021/bi7013618

[10] Karayan L, Riou JF, Seite P, Migeon J, Cantereau A, et al (2001) Human ARF protein interacts with topoisomerase I and stimulates its activity. Oncogene 20: 836–848. 11314011 10.1038/sj.onc.1204170

[11] Pommier Y, Pourquier P, Fan Y, Strumberg D (1998) Mechanism of action of eukaryotic DNA topoisomerase I and drugs targeted to the enzyme. Biochim Biophys Acta 1400: 83–105. 9748515 10.1016/s0167-4781(98)00129-8

[12] Wang JC (1996) DNA topoisomerases. Annu Rev Biochem 65: 635–692. 8811192 10.1146/annurev.bi.65.070196.003223

[13] Ayrault O, Karayan L, Riou JF, Larsen CJ, Seite P (2003) Delineation of the domains required for physical and functional interaction of p14ARF with human topoisomerase I. Oncogene. 22: 1945–1954. 10.1038/sj.onc.1206214 12673200

[14] Bandyopadhyay K, Gjerset RA (2011) Protein kinase CK2 is a central regulator of topoisomerase I hyperphosphorylation and camptothecin sensitivity in cancer cell lines. Biochemistry 50: 704–714. 21182307 10.1021/bi101110e PMC3046806

[15] Bandyopadhyay K, Li P, Gjerset RA (2012) CK2-mediated hyperphosphorylation of topoisoemrase I targets serine 506, enhances topoisoemrase I-DNA binding, and increases cellular camptothecin sensitvity. PLoS ONE 7: e50427. 23185622 10.1371/journal.pone.0050427 PMC3503890

[16] Tsao YP, Russo A, Nyamuswa G, Silber R, Liu LF (1993) Interaction between replication forks and topoisomerase I-DNA cleavable complexes: studies in a cell-free SV40 DNA replication system. Cancer Res 53: 5908–5914. 8261402

[17] Burri N, Shaw P, Bouzourene H, Sordat I, Sordat B, et al (2001) Methylation silencing and mutations of the p14ARF and p16INK4a genes in colon cancer. Lab Invest 81: 217–229. 11232644 10.1038/labinvest.3780230

[18] Lee M, Sup Han W, Kyoung Kim O, Hee Sung S, Sun Cho M, et al (2006) Prognostic value of p16INK4a and p14ARF gene hypermethylation in human colon cancer. Pathol Res Pract 202: 415–424. 16675157 10.1016/j.prp.2005.11.011

[19] Mounawar M, Mukeria A, Le Calvez F, Hung RJ, Renard H, et al (2007) Patterns of EGFR, HER2, TP53, and KRAS mutations of p14arf expression in non-small cell lung cancers in relation to smoking history. Cancer Res 67: 5667–5672. 17575133 10.1158/0008-5472.CAN-06-4229

[20] Sanchez-Aguilera A, Sanchez-Beato M, Garcia JF, Prieto I, Pollan M, et al (2002) p14(ARF) nuclear overexpression in aggressive B-cell lymphomas is a sensor of malfunction of the common tumor suppressor pathways. Blood 99: 1411–1418. 11830494 10.1182/blood.v99.4.1411

[21] Sano T, Hikino T, Xue Q, Saito T, Kashiwabara K, et al (2000) Immunohistochemical inactivation of p14ARF concomitant with MDM2 overexpression inversely correlates with p53 overexpression in oral squamous cell carcinoma. Pathol Int 50: 709–716. 11012984 10.1046/j.1440-1827.2000.01109.x

[22] Vestey SB, Sen C, Calder CJ, Perks CM, Pignatelli M, et al (2004) p14ARF expression in invasive breast cancers and ductal carcinoma in situ–relationships to p53 and Hdm2. Breast Cancer Res 6: R571–585. 15318938 10.1186/bcr912 PMC549173

[23] Ayrault O, Andrique L, Fauvin D, Eymin B, Gazzeri S, et al (2006) Human tumor suppressor p14ARF negatively regulates rRNA transcription and inhibits UBF1 transcription factor phosphorylation. Oncogene 25: 7577–7586. 16924243 10.1038/sj.onc.1209743

[24] Kaiserman HB, Ingebritsen TS, Benbow RM (1988) Regulation of Xenopus laevis DNA topoisomerase I activity by phosphorylation in vitro. Biochemistry 27: 3216–3222. 2839226 10.1021/bi00409a014

[25] McConaughy BL, Young LS, Champoux JJ (1981) The effect of salt on the binding of the eucaryotic DNA nicking-closing enzyme to DNA and chromatin. Biochim Biophys Acta 655: 1–8. 6266479 10.1016/0005-2787(81)90059-9

[26] Liu LF, Rowe TC, Yang L, Tewey KM, Chen GL (1983) Cleavage of DNA by mammalian DNA topoisomerase II. J Biol Chem 258: 15365–15370. 6317692

[27] Reikhardt BA, Kulikova OG, Borisova GY, Aleksandrova IY, Sapronov NS (2003) Status of the “protein kinase CK2-HMG14” system in age-related amnesia in rats. Neurosci Behav Physiol 33: 799–804. 14635996 10.1023/a:1025101516128

[28] Rada B, Gardina P, Myers TG, Leto TL (2011) Reactive oxygen species mediate inflammatory cytokine release and EGFR-dependent mucin secretion in airway epithelial cells exposed to Pseudomonas pyocyanin. Mucosal Immunol 4: 158–171. 20962773 10.1038/mi.2010.62 PMC3026888

[29] Chowdhury AR, Sharma S, Mandal S, Goswami A, Mukhopadhyay S, et al (2002) Luteolin, an emerging anti-cancer flavonoid, poisons eukaryotic DNA topoisomerase I. Biochem J. 366: 653–661. 10.1042/BJ20020098 PMC1222798 12027807

[30] Martinez EJ, Owa T, Schreiber SL, Corey EJ (1999) Phthalascidin, a synthetic antitumor agent with potency and mode of action comparable to ecteinascidin 743. Proc Natl Acad Sci U S A 96: 3496–3501. 10097064 10.1073/pnas.96.7.3496 PMC22321

[31] Champoux JJ (2001) DNA topoisomerases: structure, function, and mechanism. Annu Rev Biochem 70: 369–413. 11395412 10.1146/annurev.biochem.70.1.369

[32] Coderoni S, Paparelli M, Gianfranceschi GL (1990) Role of calf thymus DNA-topoisomerase I phosphorylation on relaxation activity expression and on DNA-protein interaction. Role of DNA-topoisomerase I phosphorylation. Mol Biol Rep 14: 35–39. 2161075 10.1007/BF00422713

[33] Pommier Y, Kerrigan D, Hartman KD, Glazer RI (1990) Phosphorylation of mammalian DNA topoisomerase I and activation by protein kinase C. J Biol Chem. 265: 9418–9422. 2160979

[34] Samuels DS, Shimizu Y, Shimizu N (1989) Protein kinase C phosphorylates DNA topoisomerase I. FEBS Lett. 259: 57–60. 10.1016/0014-5793(89)81493-0 2557245

[35] Soe K, Dianov G, Nasheuer HP, Bohr VA, Grosse F, et al (2001) A human topoisomerase I cleavage complex is recognized by an additional human topisomerase I molecule in vitro. Nucleic Acids Res 29: 3195–3203. 11470877 10.1093/nar/29.15.3195 PMC55829

[36] Pourquier P, Ueng LM, Fertala J, Wang D, Park HJ, et al (1999) Induction of reversible complexes between eukaryotic DNA topoisomerase I and DNA-containing oxidative base damages. 7, 8-dihydro-8-oxoguanine and 5-hydroxycytosine. J Biol Chem 274: 8516–8523. 10085084 10.1074/jbc.274.13.8516

[37] Halazonetis TD, Gorgoulis VG, Bartek J (2008) An oncogene-induced DNA damage model for cancer development. Science 319: 1352–1355. 18323444 10.1126/science.1140735

[38] Vafa O, Wade M, Kern S, Beeche M, Pandita TK, et al (2002) c-Myc can induce DNA damage, increase reactive oxygen species, and mitigate p53 function: a mechanism for oncogene-induced genetic instability. Mol Cell 9: 1031–1044. 12049739 10.1016/s1097-2765(02)00520-8

[39] Rogakou EP, Boon C, Redon C, Bonner WM (1999) Megabase chromatin domains involved in DNA double-strand breaks in vivo. J Cell Biol 146: 905–916. 10477747 10.1083/jcb.146.5.905 PMC2169482

[40] Redinbo MR, Stewart L, Kuhn P, Champoux JJ, Hol WG (1998) Crystal structures of human topoisomerase I in covalent and noncovalent complexes with DNA. Science 279: 1504–1513. 9488644 10.1126/science.279.5356.1504

[41] Redinbo MR, Champoux JJ, Hol WG (2000) Novel insights into catalytic mechanism from a crystal structure of human topoisomerase I in complex with DNA. Biochemistry 39: 6832–6840. 10841763 10.1021/bi992690t

[42] Stewart L, Redinbo MR, Qiu X, Hol WG, Champoux JJ (1998) A model for the mechanism of human topoisomerase I. Science. 279: 1534–1541. 10.1126/science.279.5356.1534 9488652

[43] Sugimoto M, Kuo ML, Roussel MF, Sherr CJ (2003) Nucleolar Arf tumor suppressor inhibits ribosomal RNA processing. Mol Cell 11: 415–424. 12620229 10.1016/s1097-2765(03)00057-1

[44] Olsten ME, Litchfield DW (2004) Order or chaos? An evaluation of the regulation of protein kinase CK2. Biochem Cell Biol 82: 681–693. 15674436 10.1139/o04-116

[45] Daya-Makin M, Sanghera JS, Mogentale TL, Lipp M, Parchomchuk J, et al (1994) Activation of a tumor-associated protein kinase (p40TAK) and casein kinase 2 in human squamous cell carcinomas and adenocarcinomas of the lung. Cancer Res 54: 2262–2268. 7513612

[46] Faust RA, Gapany M, Tristani P, Davis A, Adams GL, et al (1996) Elevated protein kinase CK2 activity in chromatin of head and neck tumors: association with malignant transformation. Cancer Lett 101: 31–35. 8625279 10.1016/0304-3835(96)04110-9

[47] Landesman-Bollag E, Romieu-Mourez R, Song DH, Sonenshein GE, Cardiff RD, et al (2001) Protein kinase CK2 in mammary gland tumorigenesis. Oncogene 20: 3247–3257. 11423974 10.1038/sj.onc.1204411

[48] Lin KY, Tai C, Hsu JC, Li CF, Fang CL, et al (2011) Overexpression of nuclear protein kinase CK2 alpha catalytic subunit (CK2alpha) as a poor prognosticator in human colorectal cancer. PLoS One 6: e17193. 21359197 10.1371/journal.pone.0017193 PMC3040762

[49] Stalter G, Siemer S, Becht E, Ziegler M, Remberger K, et al (1994) Asymmetric expression of protein kinase CK2 subunits in human kidney tumors. Biochem Biophys Res Commun 202: 141–147. 8037705 10.1006/bbrc.1994.1904

[50] Yenice S, Davis AT, Goueli SA, Akdas A, Limas C, et al (1994) Nuclear casein kinase 2 (CK-2) activity in human normal, benign hyperplastic, and cancerous prostate. Prostate 24: 11–16. 7507238 10.1002/pros.2990240105

[51] Gapany M, Faust RA, Tawfic S, Davis A, Adams GL, et al (1995) Association of elevated protein kinase CK2 activity with aggressive behavior of squamous cell carcinoma of the head and neck. Mol Med 1: 659–666. 8529132 PMC2229971

[52] Laramas M, Pasquier D, Filhol O, Ringeisen F, Descotes JL, et al (2007) Nuclear localization of protein kinase CK2 catalytic subunit (CK2alpha) is associated with poor prognostic factors in human prostate cancer. Eur J Cancer 43: 928–934. 17267203 10.1016/j.ejca.2006.11.021

[53] O-Charoenrat P, Rusch V, Talbot SG, Sarkaria I, Viale A, et al (2004) Casein kinase II alpha subunit and C1-inhibitor are independent predictors of outcome in patients with squamous cell carcinoma of the lung. Clin Cancer Res 10: 5792–5803. 15355908 10.1158/1078-0432.CCR-03-0317

[54] Faust RA, Niehans G, Gapany M, Hoistad D, Knapp D, et al (1999) Subcellular immunolocalization of protein kinase CK2 in normal and carcinoma cells. Int J Biochem Cell Biol 31: 941–949. 10533285 10.1016/s1357-2725(99)00050-3

[55] Sherr CJ (2006) Divorcing ARF and p53: an unsettled case. Nat Rev Cancer 6: 663–673. 16915296 10.1038/nrc1954

[56] Korgaonkar C, Hagen J, Tompkins V, Frazier AA, Allamargot C, et al (2005) Nucleophosmin (B23) targets ARF to nucleoli and inhibits its function. Mol Cell Biol 25: 1258–1271. 15684379 10.1128/MCB.25.4.1258-1271.2005 PMC548001

[57] Anderson RD, Berger NA (1994) International Commission for Protection Against Environmental Mutagens and Carcinogens. Mutagenicity and carcinogenicity of topoisomerase-interactive agents. Mutat Res 309: 109–142. 7519727 10.1016/0027-5107(94)90048-5

[58] Larsen AK, Gobert C (1999) DNA topoisomerase I in oncology: Dr Jekyll or Mr Hyde? Pathol Oncol Res 5: 171–178. 10491013 10.1053/paor.1999.0209

[59] Pourquier P, Pommier Y (2001) Topoisomerase I-mediated DNA damage. Adv Cancer Res 80: 189–216. 11034544 10.1016/s0065-230x(01)80016-6

[60] Zhu J, Schiestl RH (1996) Topoisomerase I involvement in illegitimate recombination in Saccharomyces cerevisiae. Mol Cell Biol 16: 1805–1812. 8657156 10.1128/mcb.16.4.1805 PMC231167

[61] Shuman S (1989) Vaccinia DNA topoisomerase I promotes illegitimate recombination in Escherichia coli. Proc Natl Acad Sci U S A 86: 3489–3493. 2542933 10.1073/pnas.86.10.3489 PMC287163

[62] Arlt MF, Glover TW (2010) Inhibition of topoisomerase I prevents chromosome breakage at common fragile sites. DNA Repair (Amst) 9: 678–689. 20413351 10.1016/j.dnarep.2010.03.005 PMC2896008

[63] Arlt MF, Durkin SG, Ragland RL, Glover TW (2006) Common fragile sites as targets for chromosome rearrangements. DNA Repair (Amst) 5: 1126–1135. 16807141 10.1016/j.dnarep.2006.05.010

[64] Hackbarth JS, Galvez-Peralta M, Dai NT, Loegering DA, Peterson KL, et al (2008) Mitotic phosphorylation stimulates DNA relaxation activity of human topoisomerase I. J Biol Chem. 283: 16711–16722. 10.1074/jbc.M802246200 PMC2423254 18408216

