# Supplementary figures and images for: The p14ARF Alternate Reading Frame Protein Enhances DNA Binding of Topoisomerase I by Interacting with the Serine 506-Phosphorylated Core Domain
Source: PLoS One. 2013 Mar 26;8(3):e58835. doi: 10.1371/journal.pone.0058835 (PMC3608632; doi:10.1371/journal.pone.0058835)

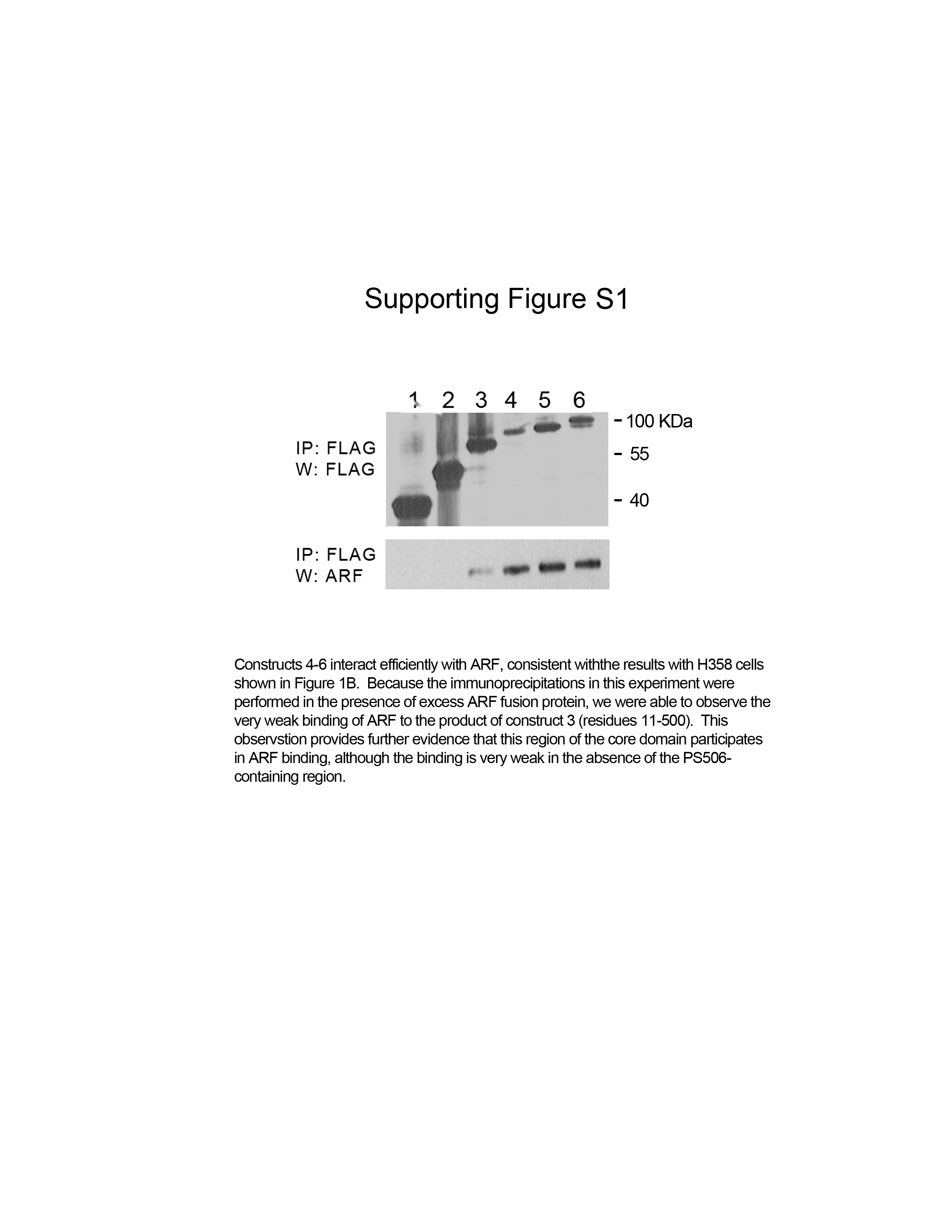

Supplement: Figure S1 — Analysis of ARF binding to the products of constructs 1–6 (see Figure 1A ) expressed in 293T immortalized human embryonic kidney cells. Two days after transfection (one 10 cm dish of cells per transfection), transduced gene products were recovered by lysing cells in high salt buffer (see Materials and Methods) followed by selection on cobalt-agarose (Thermo Scientific). The gene products were eluted in 60 µl of 1 M imidazole elution buffer (Thermo Scientific) and added to 1 ml of immunoprecipitation buffer (20 mM Tris pH 8, 150 mM NaCl, 10% glycerol, 1% NP40, 2 mM EDTA, and complete protease inhibitors. Products were mixed with 3 µg bacterially expressed recombinant human ARF and subjected to FLAG IP followed by Western analysis of FLAG or ARF, as described for Figure 1B. (TIF) [file pone.0058835.s001.tif]

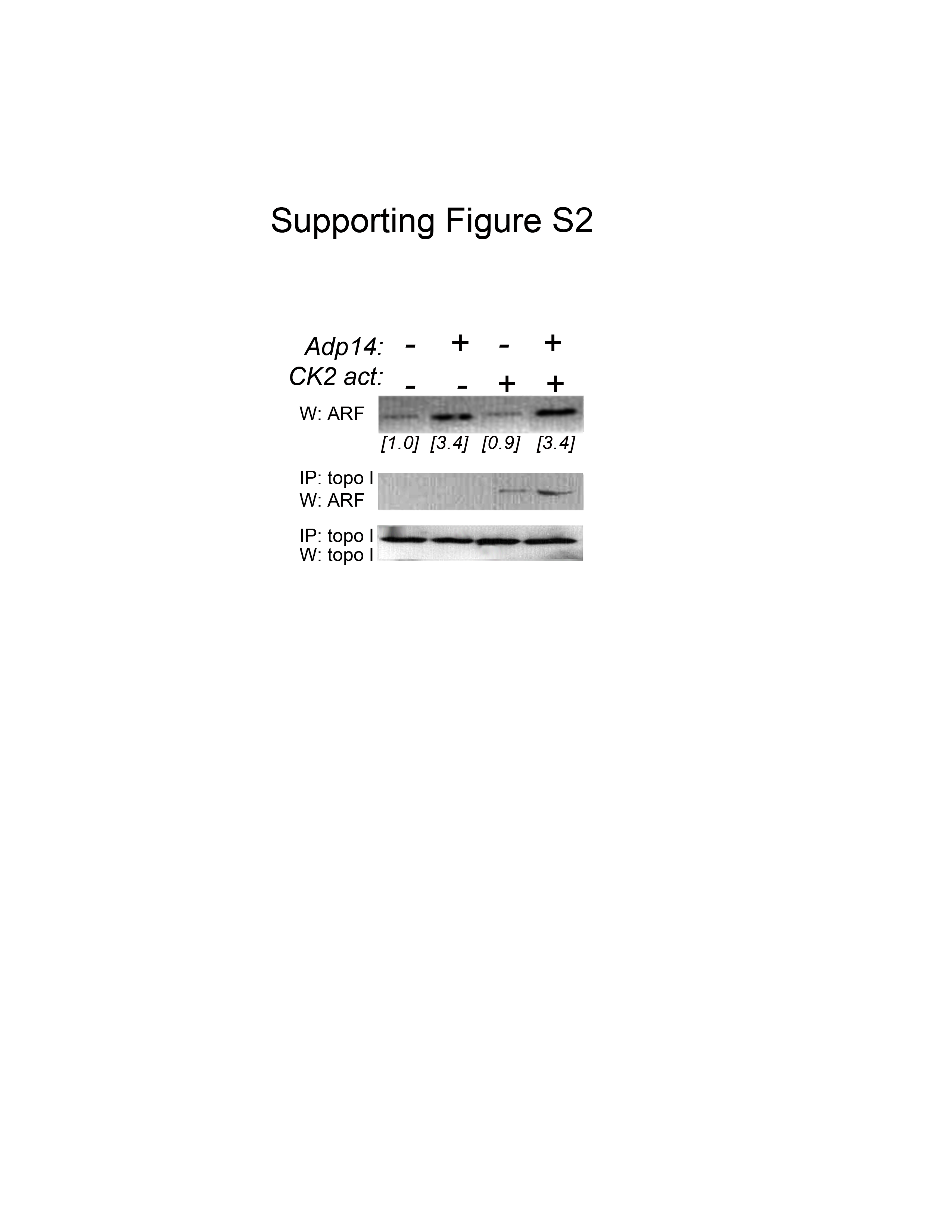

Supplement: Figure S2 — ARF–topo I complexes in OVCAR-3 cells in the presence or absence of CK2 activation and ARF overexpression. OVCAR-3 cells were treated with 20 moi of Adp14 (4 hr) with or without CK2 activator treatment (10 nM, duration of experiment). Top row: 2 days later, cells were analyzed by ARF Western to determine ARF levels. Digital quantification of bands relative to untreated is indicated in italics below lanes. Middle and Bottom rows: topo I IP followed by ARF and topo I in same samples to detect the formation of ARF–topo I complexes. (TIF) [file pone.0058835.s002.tif]

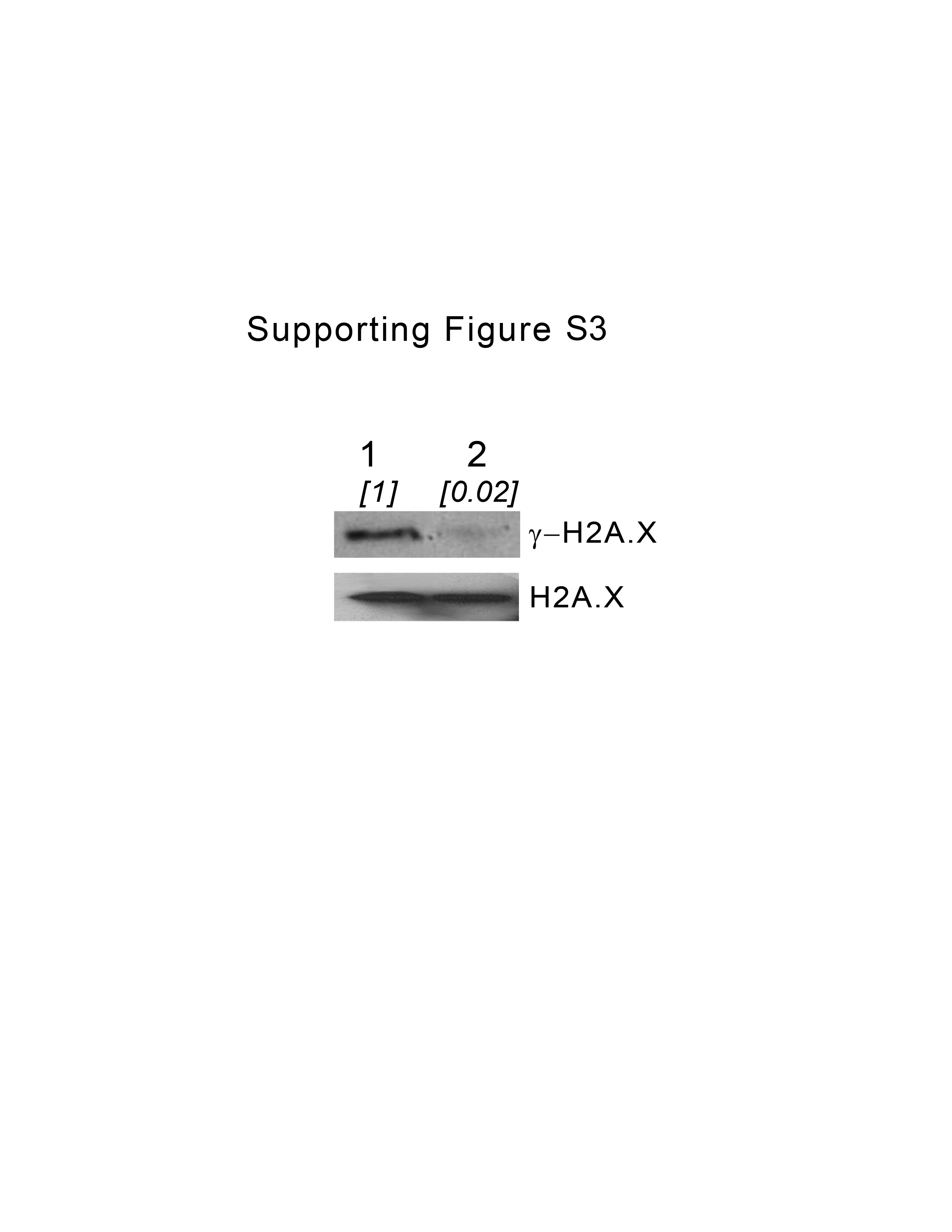

Supplement: Figure S3 — Comparison of DNA damage in OVCAR-3 cells after various treatments. Lane 1: Western analysis of γ-H2A.X and H2A.X (control) in OVCAR-3 cells, 2 days after treatment with 10 nM CK2 activator (duration of experiment) plus 80 nM camptothecin (first 18 h), a cytotoxic treatment that reduces cell viability to 20% of that observed with untreated cells [15] (lane 1); Lane 2: Western analysis of γ-H2A.X and H2A.X (control) in OVCAR-3 cells, 2 days after a combination treatment with 20 moi Adp14 (4 hours), 10 nM CK2 activator (duration of experiment) and 10 µM pyocyanin (duration of experiment). Treatment conditions same as for lane 8 of Figure 4B. Each lane represents 20 µg cellular protein. (TIF) [file pone.0058835.s003.tif]
