# Supplementary material for: The p14ARF Alternate Reading Frame Protein Enhances DNA Binding of Topoisomerase I by Interacting with the Serine 506-Phosphorylated Core Domain
Source: PLoS One. 2013 Mar 26;8(3):e58835. doi: 10.1371/journal.pone.0058835 (PMC3608632; doi:10.1371/journal.pone.0058835)
Supplement: Table S1 — Primers used for generating topo I deletion fragments. (DOCX) [file pone.0058835.s004.docx]

| Supporting Information Table S1 - Topo I primers | | |
| --- | --- | --- |
| Primers for generating deletion fragments from full length topo I in pCMV-tag | | |
| Primer: | Restriction site | sequence |
| forward from residue 11 | Bgl II | **5'- AAC AGA TCT ATC AGA TCG AAG CGG ATT TCC GAT TG -3'** |
| forward from residue 501 |  | **5'- AAC AGA TCT AT ACT GTG GGC TGCTGC TCA-3'** |
| forward from residue 216 |  | **5'- AAC AGA TCT AT AAG TGG AAA TTC CTA GAA-3'** |
| reverse from residue 215 | EcoRV | **5'- TTG ATA TCG ATG CCT TCA GGA TAG CG -3'** |
| reverse from reside 350 |  | **5'- TTG ATA TCA ATC CTC TCT TTG TGG TT -3'** |
| reverse from residue 500 |  | **5'- TTG ATA TCG TCC GCT GTT TCT CCT TC -3'** |
| reverse from residue 636 |  | **5'- TTG ATA TCG TCC GCT GTT TCT CCT TC -3'** |
| reverse from residue 713 |  | **5'- TTG ATA TCC TGT TTA TTT TCC TCT CG -3'** |
| reverse from residue 765 |  | **5'- TTG ATA TCA AAC TCA TAG TCT TCA TC -3'** |
| Primers for subcloning deletion fragments +FLAG sequence from pCMV-tag to pTriEx-2Hygro | | |
| Forward from Kozak-FLAG sequence | EcoR1 | **5’-AA GAA TTC CCG GGC GGA TCC ACC ATG-3’** |
| Reverse from myc sequence | Sal1 | **5’- CTC GAG GTC GAC GGT ATC GAT AAG -3’** |
